# Harvesting the promise of AOPs: An assessment and recommendations

**DOI:** 10.1016/j.scitotenv.2018.02.015

**Published:** 2018-07-01

**Authors:** Annamaria Carusi, Mark R. Davies, Giovanni De Grandis, Beate I. Escher, Geoff Hodges, Kenneth M.Y. Leung, Maurice Whelan, Catherine Willett, Gerald T. Ankley

**Affiliations:** a Medical Humanities Sheffield, University of Sheffield, Medical School, Beech Hill Road, Sheffield S10 2RX, UK; b QT Informatics Limited, Macclesfield SK10 5DS, UK; c Science, Technology, Engineering and Public Policy (STEaPP), Boston House, 36-37 Fitzroy Square, London W1T 6EY, UK; d UFZ – Helmholtz Centre for Environmental Research, 04318 Leipzig, Germany; e Eberhard Karls University Tübingen, Environmental Toxicology, Centre for Applied Geosciences, 72074 Tübingen, Germany; f Safety and Environmental Assurance Centre, Unilever, Colworth Science Park, Sharnbrook, Bedfordshire MK44 1LQ, UK; g The Swire Institute of Marine Science and School of Biological Sciences, The University of Hong Kong, Pokfulam, Hong Kong, China; h European Commission, Joint Research Centre (JRC), Ispra, Italy; i The Humane Society of the United States, 700 Professional Drive, Gaithersburg, MD, 20879, USA; j US Environmental Protection Agency, 6201 Congdon Blvd, Duluth, MN 55804, USA

**Keywords:** Toxicology, Adverse outcomes pathways, Risk assessment, Environmental health, Chemical management, Social, ethical and legal aspects

## Abstract

The Adverse Outcome Pathway (AOP) concept is a knowledge assembly and communication tool to facilitate the transparent translation of mechanistic information into outcomes meaningful to the regulatory assessment of chemicals. The AOP framework and associated knowledgebases (KBs) have received significant attention and use in the regulatory toxicology community. However, it is increasingly apparent that the potential stakeholder community for the AOP concept and AOP KBs is broader than scientists and regulators directly involved in chemical safety assessment. In this paper we identify and describe those stakeholders who currently—or in the future—could benefit from the application of the AOP framework and knowledge to specific problems. We also summarize the challenges faced in implementing pathway-based approaches such as the AOP framework in biological sciences, and provide a series of recommendations to meet critical needs to ensure further progression of the framework as a useful, sustainable and dependable tool supporting assessments of both human health and the environment. Although the AOP concept has the potential to significantly impact the organization and interpretation of biological information in a variety of disciplines/applications, this promise can only be fully realized through the active engagement of, and input from multiple stakeholders, requiring multi-pronged substantive long-term planning and strategies.

## Highlights

• The AOP framework aims to increase efficiency of chemical safety assessments.
• The stakeholder community for AOPs, however, is broader than chemical risk assessors.
• There are scientific and social challenges to successfully engage all stakeholders.
• Multi-faceted communication and governance strategies will address these challenges.

## Graphical abstract

**Figure:**
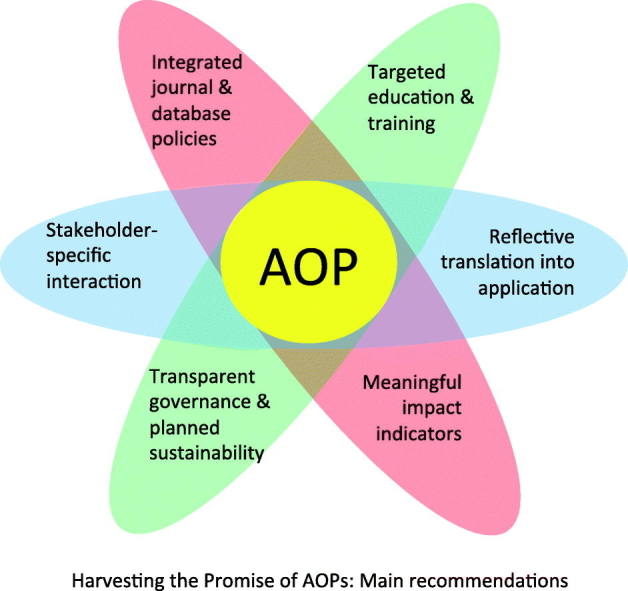

## 1 Introduction

Legislative dictates and societal expectations require a different approach to chemical safety assessment than has been used in the past when extensive data were collected for only a handful of chemicals of concern either to humans or the environment. Currently there is a need to determine the potential biological effects of tens of thousands of existing and new substances in a cost-effective and timely manner, but resources for chemical testing currently are either static or decreasing. Hence, there is increasing reliance on approaches to efficiently generate data concerning the possible biological activity of chemicals through use of in silico models, in vitro assays, and short-term in vivo tests emphasizing molecular and biochemical measures of effects (National Research Council, 2007). This creates a challenge for effectively translating results from these types of mechanistic/pathway-based analyses into information useful to those ultimately responsible for conducting chemical safety assessments.

The Adverse Outcome Pathway (AOP) conceptual framework was developed to serve as a knowledge assembly and communication tool to facilitate the transparent translation of mechanistic data into outcomes meaningful to chemical safety assessment (Ankley et al., 2010). Specifically, the AOP framework facilitates the identification and evaluation of causal linkages across biological levels of organization, such that mechanistic responses can be reliably used in decision-making to protect from adverse effects, such as cancer in humans or suppression of reproduction of ecologically important species. Many regulatory agencies across the world have recognized the potential of AOPs in supporting more efficient assessments of chemical safety. One notable example involves the Organization for Economic Cooperation and Development (OECD). The OECD initiated an international AOP development program in 2012 that has sponsored and contributed to a number of critical activities, including the publication of standardized approaches for AOP development and review, and the formation of an AOP-knowledgebase (AOP-KB) to support AOP development and dissemination (Villeneuve et al., 2014a, Villeneuve et al., 2014b; Vinken et al., 2017). A critical motivation for OECD involvement was the recognition by member countries that a pathway-based approach to chemical safety can promote economic growth as well as the highest levels of health and environmental protection (Perkins et al., 2015).

**Fig. 1:**
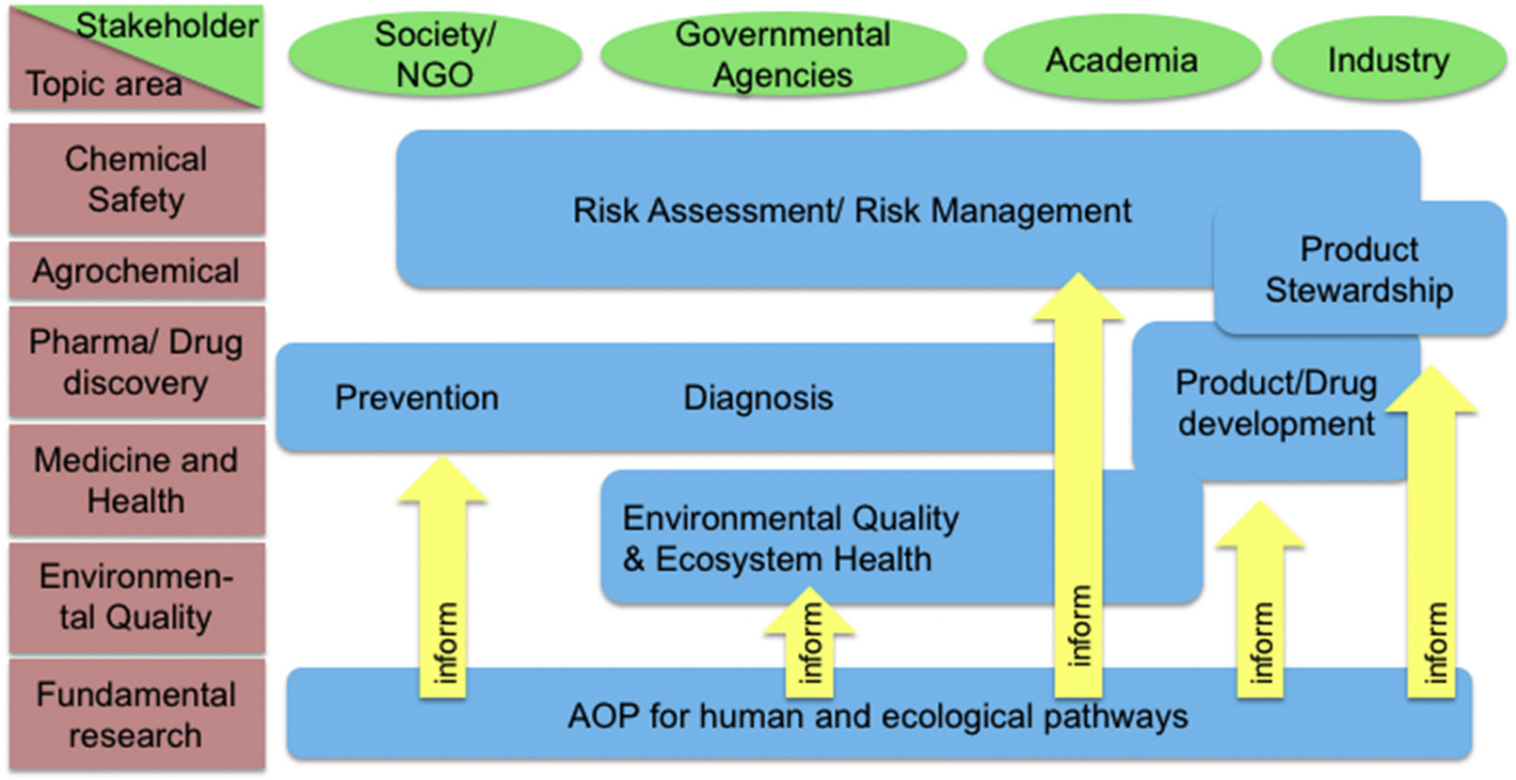
Stakeholders involved in the different areas of research and development in relation to AOP development and usage.

As the AOP framework has evolved in the context of regulatory toxicology, it has become recognized that the potential stakeholder community is broader than those involved in chemical safety assessment, as illustrated in Fig. 1. For example, the framework may complement activities to apply the tractability of mechanism-based approaches for addressing biomedical challenges (Benson, 2015; van Hasselt and van der Graaf, 2015; Visser et al., 2014), in safety evaluations associated with drug development (Mirams et al., 2014), systems toxicology (Stahl et al., 2015) and clinical trial simulations (Han et al., 2016).

Our aim with this paper is to present a strategic vision to help ensure that the AOP framework can realize its full potential for multiple stakeholder groups. The paper first details the unique features and qualities of the AOP approach and of the AOP framework, and goes on to profile a variety of stakeholders, describing how they stand to benefit from the further development of the AOP framework and its application to solving their problems. Our analysis includes consideration of the core obstacles to a full, long-term engagement of the stakeholder community with the framework. Finally, we conclude with a set of recommendations regarding the steps that need to be taken to address these challenges, and to ensure development of the AOP framework as a sustainable tool for the application of our scientific knowledge of living systems in numerous decision-making contexts.

This paper stems from a Pellston workshop held April 2–6, 2017 in Cornwall, ON, Canada. The workshop was coordinated by the Society of Environmental Toxicology and Chemistry (SETAC), and financially supported by multiple governmental and business organizations. Pellston workshops typically assemble 30–40 (invited) experts on a given topic in the environmental sciences, who are charged with defining and proposing paths forward to address challenge(s) in the topic area of interest. This Pellston workshop focused on a number of issues associated with AOP science and implementation, including the development of a “road map” to promote sustainable use of the concept. Specific needs addressed at the workshop were identified through a global horizon scanning effort that gathered input from scientists and risk assessors/managers from throughout the world (LaLone et al., 2017a). In all, 41 experts representing government, academia and business, from nine different countries in North America, Europe and Asia participated in the workshop.

## 2 Features of the AOP framework and its AOP knowledge base

Over the past several decades the global scientific academic, pharmaceutical, chemical and personal care product communities, as well as government agencies, have invested billions of (US) dollars generating biological information. The goal has been both to extend basic scientific knowledge of how biological processes causally unfold and to inform the development of less harmful chemicals and more effective disease diagnosis, prevention and treatment. Traditionally, this information has been deposited in scientific publications, but the sheer number of papers, compounded by the fact that the information is often conveyed in unstructured and inconsistent ways, makes it difficult to access, share and integrate. Initiatives to summarize and compile this information into centralized data repositories have been only partially successful, as they i) do not include all publications, ii) remain compartmentalized, and iii) use different ontologies. These disconnected databases have resulted in the next level of “silo-ed” information. The rise in bioinformatics, including collections of “omics” information and associated relational databases, has also produced a vast amount of potentially useful, but still ultimately difficult-to-access information. An additional complication is that lack of curation makes it difficult to assess the quality of much information (Tripathi et al., 2016; Lægreid and Kuiper, 2015; Howe et al., 2008).

**Fig. 2:**
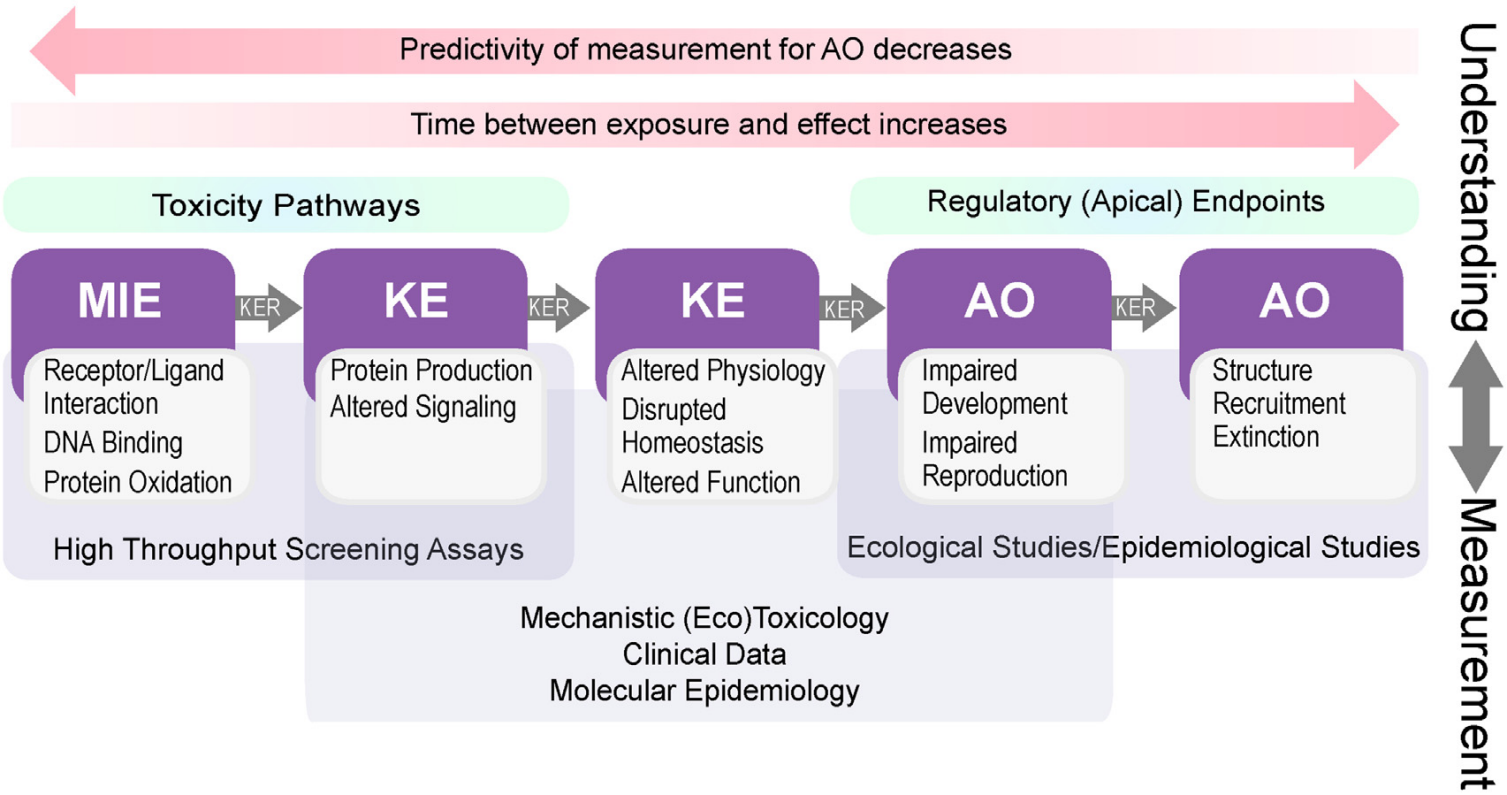
Depiction of the role of the Adverse Outcome Pathway (AOP) framework in linking various data streams to outcomes relevant to regulatory decision-making for chemicals. MIE – molecular initiating event, KE – key event, KER – key event relationship, AO – adverse outcome.

Source: From Ankley and Edwards (2018).

The goal of the AOP framework is to compile and synthesize this wealth of biological information such that it can be transparently and efficiently employed for decision-making. Fig. 2 provides an overview of the AOP framework in the context of its potential application to the translation and use of different types of data to support assessment of the effects of chemicals on human health and the environment. The initial interaction of a chemical with a biological system is depicted as the molecular initiating event (MIE), such as binding to a protein (e.g., receptors, enzymes) or DNA, or interactions with membrane lipids. These MIEs can cause subsequent perturbations at higher biological levels of organization, depicted as intermediate key events (KEs) along an AOP, which ultimately may result in adverse apical responses such effects on survival, reproduction, carcinogenesis, etc. In the case of ecological assessments, impacts at the population level also often are an endpoint of regulatory concern. Implicit in an AOP is that the depicted KEs are causally-associated with one another via defined KE relationships, an attribute that can be assessed using weight-of-evidence analyses (Becker et al., 2015). It is this documented, formalized linkage across biological levels of organization in an AOP that provides the basis for the use of “alternative” data streams to predict the types of apical responses deemed critical to regulations and risk assessments.

The AOP framework potentially goes beyond biological pathways, but will improve our understanding of chemical toxicity in relation to the ecological impacts of chemical contaminants in air, water, soil and food as well as impacts to human health. Escher et al. (2017) give a detailed account of how the mechanistic understanding of the AOP framework can be useful beyond human health for broader ecological issues. The AOP framework has wide implications for advancing the assessment of chemical hazards across various environmental compartments covering atmosphere, hydrosphere, biosphere, lithosphere, and anthroposphere.

The AOP-KB also has specifically been designed to address the problem of unstructured and scattered information. The AOP-KB currently consists of several software packages, all of which deal with collecting, formatting and, to some extent evaluating, biological information. At the time of writing, the AOP-Wiki is the most prominent and well-developed of the packages and therefore it is the illustrative focus in this paper; however, the needs and issues for engagement with the other elements of the AOP-KB will likely be similar. The AOP-Wiki is an open-source platform for collecting and organizing biological information. At present the AOP-KB contains about 223 AOPs applicable to assessments focused on both human health and the environment (http://aopkb.org, accessed 20 Sep 2017). Other AOP-KB packages include Effectopedia, AOP Explorer, and the Intermediate Effects Data Base (Table 1). A unique feature of the AOP-KB is that each of the modules is being developed and managed by different agencies or entities, but the overall organization and supporting documents are centralized under the auspices of the OECD. It is not, however, the only database for AOPs, since there will soon be a standard reporting template that can be used to deposit AOPs in a network of interoperable databases or knowledge bases, with the AOP-KB as a central hub.

**Table 1:** Components of the AOP knowledge base (KB) (http://aopkb.org/, accessed 20 Sep 2017).

Module	Description	Developing entity	URL
e.AOP.Portal	Main entry point for the AOP-knowledge base (AOP-KB)	OECD	http://aopkb.org/
AOP Wiki	An open-source platform for collecting and organizing biological information	US Environmental Protection Agency Office of Research and Development	http://www.oecd.org/chemicalsafety/testing/adverse-outcome-pathways-molecular-screening-and-toxicogenomics.htm
Effectopedia	An open-knowledge and structured platform able to display quantitative information on Adverse Outcome Pathways (AOPs)	OECD	https://www.effectopedia.org/
AOP Explorer	AOP network visualization and analysis tool	US Army Corps of Engineers	https://github.com/DataSciBurgoon/aopxiv
Intermediate Effects Data Base	Repository for key event information	European Commission Joint Research Centre	aopkb.org

Stakeholders need to be aware of all the unique features of the AOP framework that make it worthwhile for them to fully embrace it and motivate them to actively engage with the broader AOP community. At its core, the AOP framework is a comprehensive means of gathering, integrating, curating, sharing, reviewing and disseminating knowledge about the mechanisms and consequences of perturbation of normal biological function by chemical or non-chemical stressors in different organisms. Ultimately there are three “selling points” of the AOP framework, namely; i) the AOP knowledge itself, ii) the way the knowledge is assembled/treated and iii) the potential applications and impact the knowledge can have. The primary type of knowledge captured by the framework is obviously related to toxicological processes and mechanisms. However, in a description of an AOP there is also valuable information on test methods and models that can be used to measure or predict the KEs that comprise an AOP, and information on chemical initiators and their relevant chemical properties linked to the MIE. Thus when one describes the type of knowledge covered by the AOP framework, it is important to highlight the availability of information on methods and chemicals associated with measurement of the KEs.

**Fig. 3:**
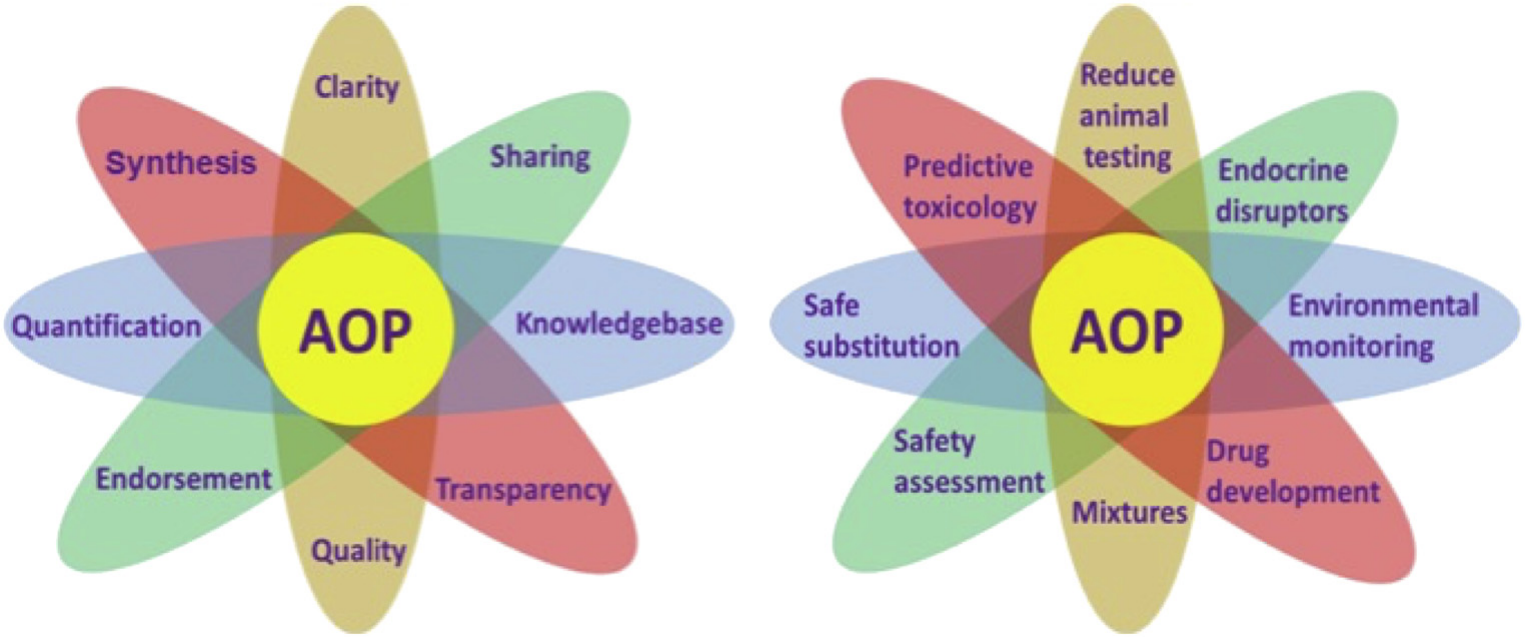
An illustration of (a) the attributes of the AOP framework and (b) the potential applications of AOPs.

Some of the key attributes of the way knowledge is managed within the AOP framework are depicted in Fig. 3(a). They include:

*Clarity* – the description of an AOP is highly structured and follows a particular format and well defined conventions and guidance (Villeneuve et al., 2014a, Villeneuve et al., 2014b; OECD, Organization for Economic Co-operation and Development, 2017, OECD, Organisation for Economic Co-operation and Development, 2016a, OECD, Organisation for Economic Co-operation and Development, 2016b, OECD, Organisation for Economic Co-operation and Development, 2016c). Once users become familiar with the format, it provides clarity, ease and efficiency when accessing AOP knowledge and ultimately provides a de facto reporting standard.

*Synthesis* – there is a wealth of existing knowledge relevant to perturbed biological pathways spread across many sources. An AOP typically captures knowledge from a large number of peer-reviewed papers and integrates and distills it into a concise form, resulting in considerable saving in time and effort for users.

*Sharing* – the framework is built with crowdsourcing and knowledge-sharing very much in mind, not only for the initial development of an AOP but also during ongoing refinement and review. Since an AOP covers many levels of biological organization, this naturally stimulates and relies upon extensive collaboration across numerous scientific disciplines

*Knowledgebase* – the most important aspect of the framework is providing ready access to AOP content. Further, with the anticipated adoption of an OECD harmonised template for reporting AOPs and the recent launch of the e.AOP.Portal (Table 1), it is possible to envision interoperable AOP platforms maintained by different parties across the world.

*Transparency* – AOP development and review processes are iterative in nature and full transparency helps to engage potential contributors directly, either as co-developers or reviewers, or indirectly through, for example, the posting of comments on the AOP-Wiki discussion pages. The identity of developers, contributors and reviews are also displayed within the AOP-KB.

*Quality* – the quality of an AOP is assessed and assured through several steps: the availability of detailed guidance for developers (OECD, Organization for Economic Co-operation and Development, 2017, OECD, Organisation for Economic Co-operation and Development, 2016a, OECD, Organisation for Economic Co-operation and Development, 2016b, OECD, Organisation for Economic Co-operation and Development, 2016c), continuous crowdsourced peer review, and curation by scientists involved in maintaining the AOP-KB. Moreover, AOPs developed within the context of OECD projects are subject to two rounds of rigorous expert review, one by an OECD expert advisory group, and the other by a panel of independent international experts.

*Endorsement* – once an AOP has successfully passed through expert review within the OECD process, it can be submitted to the Working Party of National Coordinators for the Test Guidelines Programme and the Working Party for Hazard Assessment for their collective endorsement, leading ultimately to publication of the AOP in the recently created series dedicated to AOPs (OECD, 2016c).

*Quantification* – the description of an AOP can be enhanced by the inclusion of quantitative data, such as the dynamics underpinning KE relationships (Villeneuve et al., 2014a; Wittwehr et al., 2017). In addition, more complex processes can be represented through the weighted combination of a set of interconnected AOPs within a network (e.g., LaLone et al., 2017b).

The AOP framework can be viewed by stakeholders from the perspective of its different potential applications (Fig. 3b). Some of these are quite closely related, such as reducing animal testing, predictive toxicology and safety assessment. Essentially, AOPs provide a mechanistic blueprint to design integrated assessment approaches based on combinations of in vitro and in silico methods that predict toxicological effects of interest, thus reducing the need for animal testing (Tollefsen et al., 2014). Moreover, including species-specific knowledge within AOPs helps to address the increasing demand for species-relevant assessments. Mechanistic profiling of molecular libraries during the drug development process can be effectively informed by AOP knowledge to guide the selection of appropriate batteries of in vitro high-throughput screening (HTS) assays for fingerprinting the effects of chemicals to support their safety assessment. HTS bioassays can be employed to characterise not only single chemicals but also be used to test complex environmental samples in environmental monitoring (Schroeder et al., 2016). By using concepts of mixture toxicity and effect-directed analysis, it is even possible to relate the effects observed in the complex environmental samples to known chemicals and to characterise the effects of mixtures of unknown chemicals (Brack et al., 2016; Neale et al., 2015).

The AOP framework can also be described relative to current regulatory challenges and political concerns (Fig. 3b). Many agencies worldwide are under pressure to identify and regulate endocrine disruptors are looking to AOP-based approaches for hazard and risk assessment that can help fill data gaps within a reasonable timeframe, at an acceptable cost, and keeping animal testing to a minimum (Coady et al., 2017; Browne et al., 2017). The effects of “real-life” chemical mixtures represent another considerable challenge for risk assessors. In this context, AOP networks could prove to be a vital tool to explore mixture effects in order to focus attention on chemicals and AOPs that really matter (Villeneuve et al., 2014a, Villeneuve et al., 2014b). Finally, the safe substitution of hazardous chemicals in products with less hazardous alternatives is an area of growing importance, but the use of traditional toxicity testing methods to inform the process are often too slow, ineffective and expensive. Thus, AOP-based hazard profiling and ranking potential substitutes based on their relative toxicity would be of great value to many companies operating in a variety of sectors.

## 3 Stakeholders in the evolution of the AOP framework

The AOP approach cannot deliver on its full promise without a critical mass of stakeholders, representing different disciplines and sectors, engaging it in different ways. There is a wide range of existing and potential stakeholders in the framework (Fig. 1). In this section, we profile seven areas and four groups of stakeholders who are already engaged (or could easily become more engaged) with the AOP framework. Each profile identifies a typical community of stakeholders, outlining why they are (or would be) interested in AOPs and how they could further develop the framework. The list is not exhaustive or definitive but is meant to serve as an initial analysis of potential stakeholders and their roles for the advancement of the AOP framework.

### 3.1 Regulatory assessment of chemical risks

A principal stakeholder group for the AOP framework and associated knowledge consists of regulatory toxicologists, risk assessors and managers directly involved in evaluating chemical safety and implementing risk mitigation measures. The responsibilities and needs within this group can be very diverse, depending upon jurisdiction, policy context and legislative mandate. Regulatory toxicologists and risk assessors have to deal with varied protection targets (e.g., various human groups or facets of the environment), different hazard/risk assessment scenarios, varying amounts of available data and/or levels of data collection capabilities (ranging from very little for most industrial chemicals, to large amounts for pesticides) and differing numbers of chemicals for which they are responsible (a few ranging up to thousands). Risk managers then have to use the output of a risk assessment to make decisions on whether measures should be taken as required by relevant laws and associated protection goals. Decisions might include restricting the use of a hazardous chemical or requiring its prior authorisation, and often include consideration of socio-economic consequences of intervention, which can be considerable. Accordingly, the utility and perception of value of the AOP framework will vary for different members of this stakeholder community. For example, assessors responsible for rapid processing of large numbers of chemicals with limited data may rely upon the AOP framework for the utilization of in silico and/or in vitro data for hazard profiling, which would serve as the basis for identifying chemicals of potential concern and prioritizing them for further testing or assessment. This scenario is exemplified by regulators involved in the US Environmental Protection Agency's (EPA)'s endocrine disruptor screening program, who use the AOP framework as a basis for prioritizing chemicals for possible in vivo testing based on their endocrine activity (Coady et al., 2017; Browne et al., 2017). Another scenario – such as in pesticide risk assessment – may involve members of a stakeholder group requesting the generation of new data, targeting tests/endpoints most likely to be sensitive to a given chemical. In this case, AOP knowledge can serve as a basis for guiding and optimizing test selection.

### 3.2 Chemical safety assessment for industry

The chemical industry is required to provide information for specific apical endpoints relevant to human health and the environment to support safety assessments, as laid out under various regulations such as TSCA in the US (https://www.epa.gov/tsca-inventory, accessed 20 Sep 2017), REACH Regulation in the EU (European Parliament, 2006), and China REACH (Lau et al., 2012; Wang et al., 2012; CRS, 2014). These in turn are supported by guidance documents outlining the acceptability of methods concerning data requirements. These regulatory requirements are particularly relevant for companies involved in the development of new chemicals/products, but can vary according to industry sector (e.g., personal and homecare products, solvents, etc.). However, there are also a number of other safety requirements within industry that are driven by stewardship. These include identifying chemical alternatives that have reduced environmental or human health impacts (National Academy of Sciences, 2014), enhancing approaches for chemical read-across and categorisation (chemical grouping and extrapolation between chemicals), performing strategic/targeted testing (data-gap filling), conducting biological read-across (interspecies extrapolation), identifying non-animal approaches to safety assessment, and conducting post-production environmental monitoring.

Perhaps the greatest contribution that an AOP-based approach can provide to support regulatory toxicology and risk assessment is a reduction of the uncertainty in understanding hazardous effects for safety assessment (and hence increased confidence in the safety assessment) through a better understanding of pathway plausibility and biological read-across for targeted data-gap filling, etc. (Perkins et al., 2015). In the short term the unique AOP construct allows information on KEs and KE relationship-derived causal links from widely-varying data sources (ranging from in silico to in vivo) to enable supporting weight of evidence approaches to be used in, for example, biological read-across, without the need for apical endpoint testing (e.g., Burden et al., 2015).

In the mid to longer term, the AOP approach has the potential to be used to help identify relevant pathways in the derivation of biological pathway altering doses (Judson et al., 2011) or for helping to define biological points of departure (Bercu et al., 2016), as part of the risk assessment paradigm shift outlined by the National Academy of Sciences in the US (NAS, 2014). One high-profile example of the use of AOPs in chemical safety assessment in this forward-looking manner has been in collating scientific data supporting an AOP for skin sensitization for the purpose of assessing the risk of allergic reaction in humans without the necessity of animal testing (Maxwell et al., 2014; OECD, 2012; MacKay et al., 2013).

### 3.3 Product discovery and development

The pharmaceutical (both human and veterinary) and agrochemical industries are primarily focussed on developing novel chemical entities for the purpose of medicinal and agricultural usage. Here a requirement is to efficiently identify compounds that may be harmful to human, animal health or the environment. The motivations are significant, as a number of chemicals used, for example, in commonly prescribed medications may be hazardous to the environment (see review by Daughton, 2003). A complementary need is the necessity to retain chemicals of proven therapeutic effectiveness. There are a number of opportunities to use the AOP framework to support these activities such as enriching the triaging process identifying undesirable chemical/pharmacological properties applied early in drug discovery to deprioritize chemicals likely to cause harm. Secondly, AOPs from the AOP-KB could act as a precursor for onward, more quantitative approaches based on systems toxicology (Sturla et al., 2014) for simulating the potential consequences on adverse outcomes that are still poorly characterised. For some types of adverse effects such as hepatotoxicity (Woodhead et al., 2017) and cardiac toxicity (Davies et al., 2016), there have already been demonstrations of using in silico approaches in the pharmaceutical industry. However, there is scope for extending consideration of these toxicities in support of preclinical safety assessment of drug candidates on sensorial systems, such as drug-induced disturbances on taste and smell or sleep disorders (Cavero and Holzgrefe, 2015; Cavero and Holzgrefe, 2017). Wider employment of the AOP framework to complement nascent approaches offers tangible benefits in being able to enrich the available chemistry for compounds that would be subsequently less likely to fail due to unforeseen toxicities or environmental harm.

### 3.4 Medicine and health

The AOP framework would be enriched by the knowledge and expertise of clinicians, who could contribute understanding of the biological and physiological context of AOPs, and therefore of their plausibility or potential gaps. In turn, the AOP framework is applicable to understanding disease pathways across multiple biological levels and is a highly useful addition to the “toolbox” of approaches increasingly being used in the clinic for prevention, diagnosis and treatment, and in biomedical and clinical research for drug discovery, efficacy and safety testing (Langley et al., 2015; EFPIA MID3 Workgroup et al., 2016). Clinicians are important for fully developing the biomedical potential of the AOP framework, as they could increase access to human-based data (Rodriguez et al., 2015), and give a more contextualised understanding of in vitro assays. The framework could also offer unique forms of support for clinicians using tools to ensure that prescribing is optimised for the patient. To this end, a number of decision support systems exist to help the clinician better tailor prescribed medicines that do not give rise to unintended adverse effects, such as co-medication error. Longer-term ambitions may include decision support systems that are tailored to individual patient characteristics beyond co-medications, such as diet, weight, sex and lifestyle. One such example is the CredibleMeds database (Schwartz and Woosley, 2016), which provides easy-to-use guidance to the clinician on the propensity for a particular medication to cause proarrhythmia, particularly when there are predisposing genetic factors. Whereas decision support systems in clinical contexts currently are largely based on statistical and epidemiological correlations, the AOP framework and AOP-KB would augment decision support by adding the layer of mechanistic data, making the biological basis for outcomes more transparent. This development would fit consistently with the current aspiration towards Personalised Medicine (e.g., pharmacogenomics), but would have to take heed of the challenges that such attempts encounter at the level of clinical implementation (Hedgecoe, 2004; Day et al., 2017).

The AOP framework also complements and supports the representation of disease progression, often referred to in medicine as the “natural history of disease”. This might be particularly relevant to the identification of diagnostic markers of disease onset/progression, which could essentially correspond to discrete KEs within an AOP. Future development would be particularly relevant for rare diseases, where progression is often poorly understood (FDA, 2015). Given the maturity of the AOP-based assessment for the likelihood of chemicals to cause skin sensitisation, this may substantiate the hope for further implementable system for clinician decision support.

### 3.5 Environmental quality

**Fig. 4:**
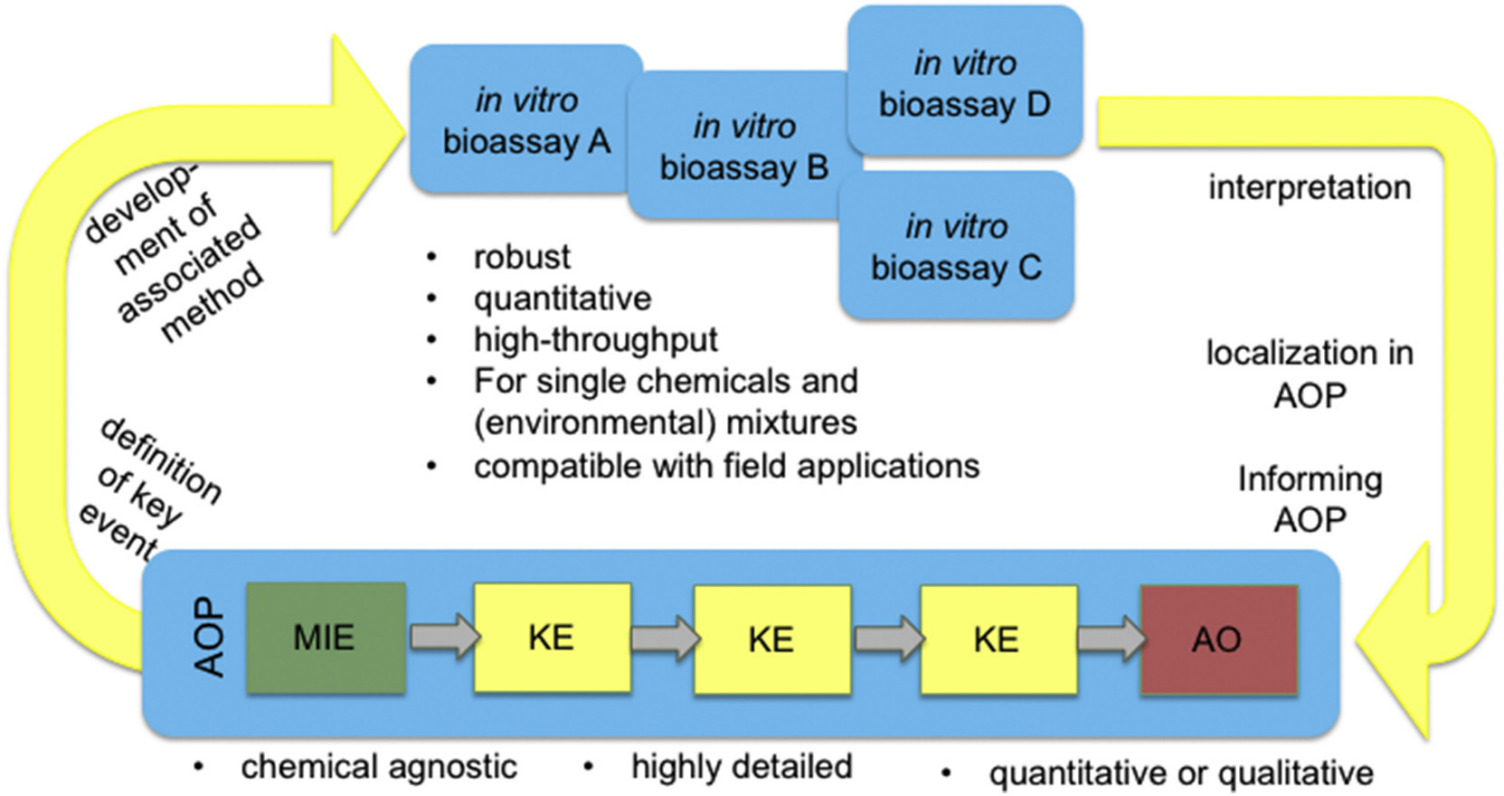
Application of in vitro bioassays for environmental quality surveillance monitoring that are anchored in the AOP framework.

Similar stakeholder groups as in chemical risk assessment are responsible for assessing the effects of chemical mixtures and complex environmental mixtures in the environment- in water and in food. Complex environmental mixtures pose a challenge that can be addressed more systematically through the application of AOP network concepts (Villeneuve et al., 2014a, Villeneuve et al., 2014b). In vitro bioassays increasingly are being used for environmental monitoring to process many samples in a time- and cost-effective manner. Recently it has been suggested that in vitro HTS assays could provide water-monitoring programs focused either on human health (e.g., drinking water) or ecological (e.g., effluent discharge) effects (Schroeder et al., 2016). The main criteria for an in vitro assay to be applicable for monitoring complex environmental mixtures is that it sensitively detects chemicals of environmental concern, and that it results in a measurement that can be clearly related to relevant apical responses in organisms potentially exposed to the contaminant mixtures (Fig. 4). The AOP concept therefore can serve as justification for the choice of in vitro bioanalytical tools (Escher et al., 2014; Neale et al., 2017). By anchoring in vitro assay results (which typically reflect MIEs but in some cases also intermediate KEs) to relevant AOPs, this information is provided efficiently and, in turn, it is possible to develop new in vitro methods from knowledge of environmentally relevant AOPs (Fig. 4).

### 3.6 Academic applications

Academic scientists currently involved with the AOP framework are mostly human health and environmental toxicologists, biochemists, molecular and cell biologists, pharmacologists and bioinformaticians. An example group of academics who have adopted the AOP concept are environmental toxicologists, who have pursued different facets of mechanistic research for many decades (Hermens et al., 1985; McKim et al., 1987; van Welie et al., 1992; van Wenzel and Opperhuizen, 1995; Escher and Hermens, 2002), but with the advent of the AOP concept were able to assemble this past work in the context of a unifying approach and common language.

Academic researchers have dual incentives as stakeholders for the AOP framework: i) they have vocational motivations as scientists who aspire to advancing knowledge in their field and teaching students; and ii) they have career motivations. For the latter, publications are a main indicator of scientific productivity and performance, and necessary for career progression in terms of positions, resources, and promotions. While there is some variability depending on country and institutions, in general there is increasing pressure on academics to demonstrate the impact of their research beyond academia. If the importance of impact on policy-making and industrial innovation continue to grow, it could provide an important incentive to contribute to AOP development.

Academic scientists are in a critical position to make a significant contribution to the uptake and establishment of the AOP framework through training new generations of researchers and graduates who will be employed in industry, policy and regulation. For example, academics are able to promote AOPs via course curricula and incorporating the AOP-KB and applications in toxicology and pharmacology courses. As exemplified in a teaching monograph by Escher and Leusch (2012) that heavily relies on the AOP framework, textbooks can greatly contribute to propagation of the AOP approach.

An example of engagement of academic stakeholders with the AOP framework is the large European research consortium project SOLUTIONS (Brack et al., 2015), which has fully adopted AOPs as a unifying concept to address the challenges related to identifying and assessing water pollution. SOLUTIONS is an EU-funded research program with the goal of developing tools for the identification, prioritisation and assessment of water contaminants that may pose risks both to ecosystems and human health, to support the EU Water Framework Directive (Brack et al., 2017; European Parliament and European Council, 2000). SOLUTIONS relies heavily on the application of in vitro whole-organism and cell-based bioassays within an AOP framework for assessing the effects of complex mixtures of aquatic contaminants (Altenburger et al., 2015). Another prominent example of a major research consortium adopting the AOP framework, this time in the human health arena, is the EU Horizon2020 funded project, EU-ToxRisk (www.eu-toxrisk.eu). The project aims to employ non-animal methods to develop new approaches to assessing the safety of chemicals in a variety of sectors, addressing in particular their potential chronic, developmental and reproductive effects.

### 3.7 Non-governmental organizations (NGOs)

NGOs give voice to concerns emerging from civil society and to the interests of those who are affected by chemical safety assessments and resulting decisions. Apart from this common goal, individual NGOs typically have differing, sometimes contrasting interests, and therefore have different incentives for engagement. Despite this, they are potential users of AOP knowledge and participants in developing and using the AOP framework.

Engagement of the animal welfare NGOs stems from the desire to move away from the use of animals for chemical evaluation and biological research (Taylor et al., 2008). A companion concern is for improved human and environmental health, but the main focus is on the animals used in laboratories. Several animal welfare NGOs have recognized the promise of AOPs, and are participating in and supporting their development (see, for example, Sullivan et al., 2017) and promotion through education, training, and dissemination (see Table 3). These NGOs are also involved in promoting the AOP framework to the biomedical community (Langley et al., 2015, Langley et al., 2016).

As environmental NGOs' core mission is to foster environmental protection, they are primarily concerned about the effects of chemicals on humans and the environment. There is a strong public concern about the large numbers of chemicals for which little safety information is available. They are also concerned about perceived and potential harm that is, or could be occurring via chemicals such as endocrine-active substances, and potential developmentally-toxic or neurotoxic chemicals (e.g., The Endocrine Disruption Exchange [TEDX], http://endocrinedisruption.org/, last accessed 20 Sep 2017). Thus far, there has been only a somewhat limited engagement of environmental NGOs, with a few notable exceptions. Specifically, since pathway-based information is used in research into the effects of pesticides on the environment (Kongsbak et al., 2014; Zhu et al., 2018) it can be expected that this will increasingly be used in the analyses of environmental NGOs, and potentially they could both participate in building AOPs, and benefit from the development and use of AOPs. At the same time, however, some environmental NGO groups have expressed concern regarding the scientific basis to the use of AOPs to support chemical safety decisions and have questioned the independence and transparency of the program at the OECD (Pesticide Action Network [PAN] EU, 2016). Increased participation of environmental NGOs could help confront these issues and facilitate their involvement in future developments.

## 4 Challenges to scaling up the AOP framework and its applications

Broad utilization of the AOP framework is dependent on involving a critical mass of stakeholders, whose participation would ensure that the framework reflects and co-evolves with their current and future needs. There are a number of challenges in ensuring that the framework adequately engages and continues to meet the immediate and long-term needs of the different stakeholders profiled above; here we identify some of these challenges and in the subsequent section recommend cross-cutting strategies that can address different aspects of these challenges (see Table 2).

**Table 2:** Multiple means for addressing the different challenges.

	Publishing and review strategy	Education and training	Stakeholder specific interaction	Translation into application	Governance and funding structures
Tensions between publication and knowledge base	x				x
Risks: financial, reputational, etc.			x	x	x
Quality control	x			x	
Cross-disciplinary/sectoral understanding		x	x	x	
Ethical, legal and social issues		x		x	x
Governance and sustainability					x

### 4.1 Formal publication versus providing input to the AOP-KB

The most significant hurdle for researchers for whom journal papers are an important output is the status of the AOP-KB relative to formal publication. Populating the AOP-KB currently requires considerable voluntary effort, and the format is not necessarily compatible with the content of peer-reviewed articles that are the basis for developing impact metrics necessary for career advancement. Prior-publication rules imposed by most scientific journals would preclude publication if the information were already made available in the AOP-KB and, once published in a peer-reviewed journal, researchers may have little incentive to deposit their data in the AOP-KB. Potential copyright infringements also may occur through uploading previously published material onto the AOP-KB (such as the Wiki) (see Section 4.5 below).

### 4.2 Possible risks and burdens to stakeholders supporting the AOP framework

While we take the view that the AOP framework is the most promising and most realistic answer to the challenges of chemical safety testing, it is necessary to highlight possible risks and burdens, so as to address them.

Policy-oriented organizations (like OECD) play a critical role in sustaining the credibility of the AOP framework and in providing assurance that AOPs can improve decision making. However, there are significant reputational risks, should there be non-optimal decision-making based on AOP information or knowledge. This risk increases as the popularity of the framework and the number of AOPs grow and the quality control process becomes itself increasingly crowdsourced and not limited to a tight community of highly dedicated scientists, as currently is the case.

Governmental and industrial organizations involved in safety assessment are key players in securing the actual usefulness and hence the ultimate success of the AOP framework. But they are also those facing the most serious risks in adopting them, since they could be held accountable for harm to consumers, the environment or business interests. There needs to be confidence that decisions based on AOP considerations are as good as conventional approaches.

Serious obstacles may come from lack of adequate incentives for developing and adopting new testing methods. For those in industry, the pressures to deliver assessments to an existing regulatory standard mean that there are few resources to consider alternative and new approaches. With significant initial development costs required to demonstrate robustness of a new approach, both in terms of intellectual and financial input, this means that often corporations are not in a position to support significant changes in an approach until it has already been sufficiently vetted by other organizations. Regulatory agencies are also resource- and time-strapped, such that adding another source of information to be weighed against other types of scientific evidence can make assessments more complicated.

### 4.3 Quality assurance

AOPs can serve a number of functions ranging from, at their most basic, assembling available data as a basis for understanding what is and what is not known about a given pathway, to relatively complete, quantitative depictions of a pathway that could be applied to risk assessment. Accordingly, Villeneuve et al. (2014a) describe a spectrum of AOP development from “putative” to “quantitative”. Becker et al. (2015) note that AOPs at all stages of this spectrum could be employed for different applications, but users need to consider the degree of their development and level of confidence in a given AOP in the context of whether it would be “fit for purpose” for a given application. Basically, the greater the impact of the application, the greater the confidence required in a supporting AOP. This requires that there be a formal process to assess AOP quality.

From a science point of view, the issue of quality control is tightly interconnected with that of publication and peer review: the “gold standard” for quality assurance of research results. Accordingly, the AOP community has established a multi-level system of reviewing well-developed AOPs, internally and externally. For example, OECD-endorsed AOPs have a level of peer review equivalent or superior to that of a published paper. OECD endorsement is recognized as bringing with it an important reputational added value, especially by the regulatory community. Career wise for the academic community, there is still a preference for publishing in known, and high impact journals, even though they may recognise that the level of peer review is equivalent. The review process is also an important way of building the AOP community, as it draws in more contributors than those who develop and upload AOPs. However, there is a question whether such a work-intensive process can be sustained in the long term, especially in consideration of the lack of incentives for some of the reviewers. An increasing number of AOPs uploaded onto the AOP-KB would also make greater demands on the pool of qualified, often over-committed reviewers.

### 4.4 Maintaining effective dialogue about the AOP framework among stakeholder groups

The AOP framework originally arose from the discipline of ecotoxicology to support regulatory decision-making (Ankley et al., 2010). This means that collaborators in other fields may not necessarily be interested in all its features, and may misunderstand aspects of the concept, or find the framework too constraining or simplified for their own needs. A recent survey described by LaLone et al. (2017a) identified a variety of concerns about AOPs, and attempted to address these using a “frequently asked questions” approach. For example, an issue often raised by researchers in biology, from academia and elsewhere, is the perceived linearity of the AOP, which is a misconception.

Specifically, AOPs can be assembled into ‘non-linear’ networks which at times can be a better reflection of reality and thus be a focus of application (Villeneuve et al., 2014a, Villeneuve et al., 2014b; LaLone et al., 2017b). It must be noted however, that although these provisions in AOP development allow for non-linarites and modulating factors to be included as narrative in the description of an AOP, the AOP construct itself was never intended to provide a means of representing a toxicological process as a formalized (mathematical) dynamical systems biology type model.

An important challenge in stakeholders dialogue arises because of conflicting perceptions of the utility associated with different levels of detail included in the AOP-KB. The tension between development and communication in the AOP-KB is particularly prominent for different stakeholder communities reflecting “users” as opposed to “developers”, with the former often requiring less complexity than the latter.

### 4.5 Ethical, legal and social aspects associated with use of the AOP framework

There are legal issues around intellectual property rights (IPR) in the production and dissemination of AOPs that deserve attention and action. Since many scientists need first to publish the results of their research in academic journals, steps should be taken in order to enable scientists to reuse their work for contributing to or authoring AOPs without infringing on copyright ownership. Conversely, there is a need to enable researchers to reuse parts of their research that have been uploaded on the AOP-KB in publications without violating anti-plagiarism rules. In addition, AOPs published by the OECD do not specify what kind of copyright, if any, protects the intellectual work and which uses are authorised. This is striking when intellectual work freely accessible through the Internet often comes with clearly specified licences. The same applies to what is available through the AOP Wiki, which by its nature as a wiki platform, is often associated with open access and free sharing or reuse of content. Authors need to have a clear perception of the kind of IPR protection available for work shared through the AOP-KB.

AOPs are not only a tool for systematically organizing and curating scientific knowledge and indicating knowledge gaps that need to be filled, but have been promoted and are perceived as an important instrument for regulators dealing with protecting the environment and human health. As such AOPs may be used in regulatory contexts where the stakes and uncertainty are high, fierce controversies may arise and stakeholders may become polarized, as exemplified by recent activism around endocrine-disrupting chemicals in the EU (Le Monde, 2016).

An obvious ethical issue that AOP usage brings to the fore is the widespread desire to reduce or ultimately replace animal testing for chemical safety assessment. As noted above, this is a strong motivator for animal welfare NGO stakeholders, but it may also result in tensions with other ethical priorities, for example, associated with perceptions regarding the role of animal tests in the effective protection of human health and the environment. The interplay and potential conflicts between these ethical priorities needs further analysis.

The social aspects of AOPs are related to the fact that they can be used by many different parties with different agendas and purposes, and this could lead to misuses prejudicial for human or environmental safety, or economically inefficient and burdensome. Recent trends in science policy and research funding emphasise that scientists need to make an effort to foresee and prevent the misuse of their knowledge and results (Ziman, 2001; Guston and Sarewitz, 2002; von Schomberg, 2007, von Schomberg, 2013; Stilgoe et al., 2013), as is also evident in the European Commission - Horizon 2020 (n.d.)- Science with and for Society programme (https://ec.europa.eu/programmes/horizon2020/en/h2020-section/science-and-society, accessed 20 Sep 2017). A robust consensus about the appropriate conditions and domains of application of AOPs is therefore needed. In other words, different stakeholders need to agree on when (i.e., at what level of development) AOPs are fit for what purpose (e.g., prioritisation of chemicals for testing, to make a regulatory decision, or for use as evidence in courts etc.; Becker et al., 2015). In practice, this demands attention both to the production of knowledge (making sure that AOPs meet high standards of epistemic robustness and are not influenced by research susceptible to conflicts of interests or biases) and to the translation and integration of that knowledge into social processes. Such integration also raises the question of how to deal with incompleteness of scientific knowledge and whether and how to complement it with non-scientific knowledge dispersed in society (Funtowicz and Ravetz, 1993; Nowotny et al., 2001; De Grandis, 2016).

### 4.6 Governance and sustainability

As the AOP framework continues to evolve, governance and coordination of the effort in the future would ideally involve all stakeholders as much as possible. Objectives of overall governance arguably would be best achieved through an impartial organization that broadly represents the various stakeholder groups. This could be accomplished through a dedicated professional society charged with coordinating advances in AOP knowledge and communication for the common good of all. Currently, the Society for Advancement of AOPs (SAAOP; http://www.saaop.org/, accessed 20 Sep 2017) handles several practical logistics associated with the wiki module of the AOP-KB, including funding an open-access server, and providing support to ensure that information supporting new or existing AOPs is entered into the system in a manner consistent with established OECD guidelines. However, the SAAOP is comprised of a comparatively small group of people, none of whom are primarily dedicated to AOP-associated governance or coordination. This is not a sustainable long-term situation.

Furthermore, to date, much of the investment in the basic AOP infrastructure (e.g., key software in the AOP-KB, training, educational and promotional materials, etc.) has come via resources provided from a relatively limited number of stakeholder groups, principally the US EPA and the European Commission, often under the auspices of the OECD. Since there is no assurance of continuing availability of resources from these organizations, the development of a stable, long-term “business plan” for provision of resources to cover these direct costs including dedicated personnel needs to be defined. Additionally, there needs to be a formal vision to attract and involve other institutional investors.

## 5 Recommendations

Many challenges that have been discussed in the previous section can be addressed in several ways. Solutions are proposed in this section; however, there is not always a one-to-one mapping between challenges and solutions. Instead, there are cross-cutting solutions to meet multiple challenges (Table 2).

### 5.1 Publishing, depositing and reviewing strategies

The AOP framework is part of a rapidly changing publishing infrastructure that is affecting almost all sciences (Clark, 2014). For example, new data repository-oriented journals give rise to an opportunity to facilitate the process of integration between publication of AOPs in the AOP-KB and in peer reviewed journals. In addition, many journals already allow supplementary information to be deposited in accredited repositories, instead of as pdf files downloadable only from the journal's website. A possible step forward would be to negotiate with publishing houses the acceptance of the AOP-KB as a site for supplementary information for published papers, in a manner analogous to the approach used for genomic data archived in the NCBI GEO database (https://www.ncbi.nlm.nih.gov/geo/, accessed 20 Sep 2017). For example, those involved in AOP governance could discuss the possibility of making deposits into the AOP-KB, or using the AOP framework standardized format for depositing in other databases mandatory with the publication of AOPs in journals such as *Environmental Science and Technology, Environmental Toxicology and Chemistry* and *Toxicological Sciences*, all of which publish AOP-oriented papers. An example of how this can be done is illustrated by Fay et al. (2017) who note that “The examples presented in the following sections, …, are available in the AOP knowledgebase …; AOP and KE numbers noted below reflect entries within this database. For simplicity, KER numbers (direct and indirect) are not displayed in the AOP figures; however, the web addresses for each AOP and its corresponding KEs and KERs are provided in the Supplemental Data.” There are multiple benefits to be gained from this integrative model: the researchers get credit for their publications and deposited data, the AOP data are openly accessible to peer-reviewers, and the growth of the AOP-KB and related databases would be accelerated.

Other possibilities in next generation publishing could include developing a database-only journal to complement the AOP-KB. There is an increasing trend towards database-only journals such as *Molecular Ecology Resources* (Wiley, http://onlinelibrary.wiley.com/journal/10.1111/(ISSN)1755-0998, accessed 20 Sep 2017) and *Scientific Data* (Nature, https://www.nature.com/sdata/publish, accessed 20 Sep 2017) in which the authors publish their work by depositing the research data following a stringent format requirement, and provide detailed descriptions of methods used for data collection, quality control and assurance, processing and analyses. These database-only journals provide a new framework for data sharing, thereby accelerating the pace of scientific discovery. Such journals could be sponsored by technical societies whose membership includes large numbers of AOP stakeholders, such as the Society of Environmental Toxicology and Chemistry (SETAC) or the Society of Toxicology (SOT).

Finally, it also might be possible to establish a journal whose main focus is AOPs, though this could be perceived as a relatively narrow field of science thus limiting the number of contributors and hence restricting its growth. Since the development of any new journal would take time and resources, and obtaining an ISI Impact Factor can take at least 3–5 years, this option could discourage academics from submitting to an AOP-only oriented journal during its early development phase.

For any of these solutions, it is paramount that the data format be compatible and interoperable, so that information can be added onto the AOP-KB with minimum effort. Steps to ensure this are being undertaken by the OECD.

### 5.2 Education and training: stakeholder-specific approaches and materials

Outreach to various stakeholder groups requires different strategies and educational materials. Some common and overlapping characteristics of stakeholders that will affect communication strategies include whether they are AOP developers, users of AOP-based information, or simply interested in understanding the concepts and the science behind AOPs and their use. A list of existing guidance documents and training presentations is available from SAAOP.org, but other centralized repositories for this type of information are needed.

To date, most outreach has focused on scientific training of current and potential AOP developers, and a number of materials have been created for this purpose (Table 3) Developers of AOPs need to understand the history, background and potential uses of AOPs as well as information concerning the AOP-KB. AOP developers also need detailed guidance on collecting, organizing, naming and evaluating the data that inform an AOP. There is a need to create greater awareness of the training material that is already available, and constant effort needs to be directed at creating suitable targeted material, and disseminating this to pertinent stakeholders.

**Table 3:** Examples of existing AOP training material.

Source	Content
*For developers*	
OECD	Information about AOP Approach: http://www.oecd.org/chemicalsafety/testing/adverse-outcome-pathways-molecular-screening-and-toxicogenomics.htm; Guidance documents: OECD, Organization for Economic Co-operation and Development, 2017, OECD, Organisation for Economic Co-operation and Development, 2016a, OECD, Organisation for Economic Co-operation and Development, 2016b, OECD, Organisation for Economic Co-operation and Development, 2016c
European Commission Joint Research Center	Background information on AOPs and AOP Wiki: https://eurl-ecvam.jrc.ec.europa.eu/about-ecvam/networks-and-collaborations/adverse-outcome-pathways-aop
Human Toxicology Project Consortium (HTPC) and the Physicians Committee for Responsible Medicine (PCRM)	Introductory PowerPoint presentations and videos available at https://humantoxicologyproject.org/aops-101/ More in-depth video presentations about AOPs: https://humantoxicologyproject.org/about-pathways/ or through the AOP Learning Channel https://www.youtube.com/channel/UCeP-bYPc1CRD3Ieaqrl-ysg
HTPC	Online AOP training course consisting of two modules, an introduction to AOPs and a tutorial on using the AOP Wiki. Available for download from https://humantoxicologyproject.org/about-pathways-2/aop-online-course/, or run directly from https://aopwiki.org/

*For scientists*	
HTPC and PCRM	Introductory PowerPoint presentations and videos available at https://humantoxicologyproject.org/aops-101/ More in-depth video presentations about AOPs: https://humantoxicologyproject.org/about-pathways/ or through the AOP Learning Channel https://www.youtube.com/channel/UCeP-bYPc1CRD3Ieaqrl-ysg
HPTC	AOP Online Course: https://humantoxicologyproject.org/about-pathways-2/aop-online-course/ or https://aopwiki.org/
OECD	Effectopedia Channel https://www.youtube.com/channel/UCpAWpL0TS53rtPcwYvFC55Q
PCRM	‘Adverse Outcome Pathways: Path to Improved Chemical Tests without Animals’ http://www.pcrm.org/AOPs
SETAC	http://setac.sclivelearningcenter.com/index.aspx?PID=9484&SID=215605
National Centre for the 3Rs (NCRs)	Pathways Based Approaches Resource Page: https://www.nc3rs.org.uk/pathways-based-approaches-resource-page
US Environmental Protection Agency	Adverse Outcome Pathways Research Brief: https://www.epa.gov/chemical-research/adverse-outcome-pathway-aop-research-brief

*For non-scientists*	
HTPC	Toxicity 101 (text descriptions) (https://humantoxicologyproject.org/tox-101/) Short videos (https://humantoxicologyproject.org/tox-101/video-series-pathways-to-a-better-future/). The first video, “The Future of Toxicology,” is available in English, Japanese, Korean, Mandarin and Portuguese.

Finally, as university teaching evolves from learning content to mastering problem-solving tools, the AOP concept offers an opportunity for a structured approach to environmental toxicology and ecotoxicology. Curricula may include practical courses on the development of AOPs as team-building literature review courses or may apply the conceptual framework of logic and uniform structuring of information. New venues for spreading of AOP information that remain to be explored are the emerging massive open online course (MOOC) that range from free availability to formal inclusion into universities' curricula (Paton, 2014). At writing, the online training course AOP Wiki module is in the process of becoming a certification course, which could serve as the first version of an open online course.

### 5.3 Stakeholder-specific interaction

Different stakeholder communities have different needs regarding how much information they require, how they prefer it presented, and how they most productively could interact with the AOP-KB and approach. Research shows that virtual collaborative environments require significant buy-in from the communities for which they are designed, but this depends on a number of factors, not least of which is the active participation of the communities in designing and developing the interface of the knowledgebase (Carusi and Reimer, 2010). The ‘build it and they will come’ approach does not work in engaging stakeholder communities. We recommend participant-based approaches to usability studies and an iterative approach to platform development, as this addresses two issues at the same time: the usability of the interface for the different communities, and their investment in its success through their participation in developing it.

### 5.4 Translation into application

Although AOP development principles are well described in guidance documents (OECD, 2016a) and in the scientific literature (Villeneuve et al., 2014a, Villeneuve et al., 2014b; Fay et al., 2017), the actual development of an AOP can be an extensive piece of work, equivalent to a systematic literature review. Case studies provide a very practical and powerful means of demonstrating the overall approach to AOP development and are an ideal vehicle to engage and inform relevant parties, ranging from potential scientific contributors to decision-makers interested in application. Case studies are most illustrative when the context in which the AOP is being developed is clearly described, together with explanation of the process undertaken to assemble and weigh the evidence (Fay et al., 2017; LaLone et al., 2017b; Horvat et al., 2017). Thus good, impactful case studies demonstrate the process as much as the results. To the extent possible, case studies would benefit from the active involvement both of those developing and using AOPs/AOP KBs of interest.

Case studies are also important to illustrate the application of AOPs in different chemical risk assessment contexts (ECHA, 2016) and are very complementary to general guidance (OECD, 2016b). For example, the 12 case studies published by the OECD concerning ‘defined approaches’ for determining the skin sensitisation potential of chemicals (OECD, 2016c) illustrate how the knowledge captured in one AOP (OECD, 2012) can provide the mechanistic basis for many different solutions to the same problem. Moreover, these particular case studies contributed significantly to the decision by EU regulators in 2016 to change the standard information requirements under REACH (European Parliament, 2006), requesting that in the first instance chemicals be assessed in terms of their potential to trigger KEs of the skin sensitisation AOP instead of conducting the standard animal test.

AOPs at different stages of development and backed by different levels of evidence are fit for different purposes (Becker et al., 2015). For instance, an incomplete AOP with important gaps may be very useful for a researcher interested in understanding which knowledge gaps need to be filled, but not appropriate for regulatory applications. As a tool facilitating the translation of scientific knowledge into regulatory decision making and promoting the production of the knowledge needed by regulators, the AOP-KB needs reflexivity in order to be responsive and responsible towards society. This means that the knowledge made available to several stakeholders through the AOP-KB should be presented so that each different group of stakeholders understands and interprets correctly its level of robustness and suitability for their own purposes. Reflexivity requires “being aware of the limits of knowledge and being mindful that a particular framing of an issue may not be universally held” (Stilgoe et al., 2013), and this demands that limitations recognized by the author of an AOP should be conveyed to potential users. It is important to work closely with stakeholders in developing ways of presenting AOPs that provide them with the necessary information for using them appropriately. Considering that users are not yet fully well-versed in using the AOP-KB, that misuses could have a serious impact either on the environment or human health, and that misuse leading to serious harm may discredit a novel tool with great promise in the eye of the public, we suggest establishing a *translational surveillance* exercise: an active monitoring of decisions made on the basis of AOPs. The goal would be to promptly detect and flag up inappropriate uses that can have negative consequences for safety and bring the AOP framework into disrepute. Moreover, this would provide very useful feedback for improving the communication strategy of the AOP-KB.

### 5.5 Governance and sustainability

Various alternatives are possible in terms of governance. While the role of the SAAOP could be expanded to support a broader stakeholder community, an alternative option might be to engage other professional societies with a stake in AOPs. For example, the SETAC and SOT are well-established scientific organizations representing a diverse membership, many of whom already are stakeholders in the AOP community. There would be a variety of mutual benefits to establishing a governance and coordination body for AOP-oriented activities through a multipartite SETAC/SOT consortium of some type.

To ensure sustainability and advancement of the AOP framework, adequate, ongoing financial support is a prerequisite for continuity and growth in the future. Currently, the AOP-KB (largely the AOP-Wiki) platform is supported by membership fees (from the SAAOP). While the OECD has funded the recent developments of Effectopedia and the e.AOP.Portal, the technical support and input for development of the AOP-Wiki are primarily contributed by volunteer experts. The current, very restricted, funding model will hinder the further growth of the AOP-KB, and even threatens its sustainability in its current form.

Continuous funding would be required to expand the IT hardware (e.g., servers), and human resources, such as full-time personnel, to upgrade software, enrich bioinformatics analyses, maintain the online system and coordinate/organize promotion and outreach activities with a view to supporting further growth of the AOP platform.

A critical role of a governance unit focused on AOPs would be development of a formal Business Plan, for example, a multi-year AOP Development Plan (ADP), presenting a sustainable financial budget and expense projections alongside with deliverables supporting the various stakeholder communities. Based on this ADP, a governance unit should be able to solicit resources from various potential funding agencies, charitable trusts and donors. Our vision is that this unit would be a non-profit NGO (consistent with the current SAAOP model), which in order to avoid potential conflicts of interest might impose a limitation on sources of funding. For instance, to have a governance body supported *only* by industry or depending too much on the contributions of one or a few donors would be inappropriate and undermine independence. Consequently, it would be important that contributions supporting AOP development/system maintenance come from a variety of contributors from different sectors, and that none can exercise undue pressure on account of their relative financial weight.

The GenBank (https://www.ncbi.nlm.nih.gov/genbank/, last accessed 20 Sep 2017), which is an open access, annotated database containing nucleotide sequences and their protein translations, could serve as a model for AOP governance/support. Initial funding from among others the National Institutes of Health, allowed for further collaboration with private enterprise. Early successes (Benton, 1990) cemented a lasting partnership between central government and bioinformatics industry in funding the bank.

Following the GenBank example, an AOP governance body could initially seek resources from multiple sources representing the whole range of stakeholders and geographies. Since the AOP platform is an international project, the funding may come from various countries. We recommend that a nascent AOP Consortium consider development of partnerships with the United Nations Environment Program (UNEP) under their program for the Strategic Approach to International Chemicals Management (SAICM). SAICM aims to promote chemical safety around the world, and provides funding to support relevant projects. While funding is primarily directed to developing countries, SETAC's long term history of collaboration with SAICM could provide a good basis for exploring the possibility of getting support for the AOP-KB in the context of the development of the “The Strategic Approach and sound management of chemicals and waste beyond 2020” (http://www.saicm.org/Meetings/FirstIntersessional/tabid/5463/language/en-US/Default.aspx, accessed 20 Sep 2017).

Funding agencies at country or regional levels also may be interested in supporting more targeted AOP endeavours. For example, the UK-based National Centre for the Replacement, Refinement and Reduction of Animals in Research (NC3R^s^) recently sponsored a call for proposals on the development of an AOP for cardiotoxicity under their strategic award funding scheme (https://www.nc3rs.org.uk/, accessed 20 Sep 2017). In the US, one could envision that various units within the National Institute of Health could include similar disease or pathway-specific calls, or include contribution of information to the AOP Wiki as a requirement for funding, in the same way as the EU's Horizon 2020 programme has provided funding to the EU-ToxRisk consortium, (which includes a specific objective to develop AOPs for the KB).

In addition to OECD, UN and other international organizations and private companies, support for the AOP framework might be sought from charitable foundations and trusts. Prominent foundations like the Bill and Melinda Gates Foundation, the Parker Foundation have funding programs whose remit could include AOPs development; likewise support may be sought from, for instance, the PEW Charitable Trust and J.P. Morgan.

### 5.6 Impact indicators

With the desire to create and maintain momentum for the development, implementation and application of AOPs by stakeholders and to support a plan to facilitate these actions, there is a need to understand how successful the community has been at achieving its objectives. Here we recommend key impact indicators to measure success. Our criteria in selection of the key impact indicators are that they align to the objectives and should be simple to adopt and measure. If we define the overarching objectives of the strategy as i) to develop a sustainable platform for the derivation of AOPs, ii) to engage and encourage stakeholders in developing and using AOPs, and iii) to embed AOPs and AOP thinking into decision-making we propose some initial “Impact Indicators” to measure success (Table 4). Additional Impact Indicators may be adopted at later stages as the framework develops further.

**Table 4:** Possible impact indicators to measure impact and success of the AOP framework and community initiatives.

Success objective	Impact indicator	What	Timeline
1	Knowledgebase (KB) metrics	Increase in the number of Wiki and webpage hits	Baselining within 6 months + repeated annually
		Increase in the number of AOPs developed and in the KB	Baselining within 6 months + repeated annually
		Increase in the number of OECD peer reviewed AOPs	Baselining within 6 months + repeated annually
		Increase in the number of active users of the KB	Baselining within 6 months + repeated annually
	Clear sustainable governance and supported platform	A defined governance structure and an understanding of funding sources to provide technical support	Within 1 year
2	Increase in biological coverage	Increase in pathways and species AOPs	Baselining within 6 months + repeated annually
	Publications	Increase in publications related to AOPs	Baselining within 6 months + repeated biannually
	Citations	Increase in citations of AOP papers	Baselining within 6 months + repeated biannually
	Altmetrics	Increase in including (but are not limited to) citations on Wikipedia, discussions on research blogs, mainstream media coverage, mentions on social networks such as Twitter.	Baselining within 6 months + repeated annually
	Training and communications	Increase in available online training materials and hits/uses of training materials.	Baselining within 6 months and repeated biannually
	Uptake in awareness/acceptance and in formal training	Survey of awareness and use of AOPs focussed initially on key stakeholders, SETAC/SOT members and establishments teaching toxicology courses (to include students).	Baselining within 6 months and repeated in 2 years.
3	Uptake in decision making	Assessment of the number in use in regulatory submissions (REACH (European Parliament, 2006) and PMN under TOSCA) and use in Investigational new drugs (IND) applications	Assessment in 2 years of the number of applications including AOPs over the previous 2-year period.

## 6 Conclusion

Toxicological knowledge and regulation are currently at a turning point; historical approaches to regulatory toxicology are no longer scientifically, economically or socially robust enough to deal with the challenges of assessing chemicals that affect the environment, human and animal health. The AOP framework and associated knowledge has the potential to make a significant contribution to addressing these challenges, as well as parallel demands in biomedical fields. Realizing this potential is a two-pronged process: the first is to fully incorporate the AOP framework into toxicological research and related scientific fields; the second is to populate the AOP-KB with AOPs, making the constantly evolving knowledge on AOPs consistently organised and curated, accessible, shareable, and fit for purpose in regulatory and other decision-making contexts. In this review, we have identified stakeholders who both stand to benefit and to make significant contributions to the AOP approach, discussed the main challenges to full involvement with the approach, and made far-reaching suggestions for a proactive, cohesive and targeted approach to fully realizing the potential of the AOP approach.
